# Factors influencing cardiovascular system-related post-COVID-19 sequelae: A single-center cohort study

**DOI:** 10.1515/med-2024-0950

**Published:** 2024-05-10

**Authors:** Xiaoyu Zhao, Dongli Wang, Yongzhi Chen, Na Zhang, Tianshu Li, Ruixia Fan, Lei Yang, Chuanhua Yang, Jie Yang

**Affiliations:** School of Traditional Chinese Medicine, Shandong University of Traditional Chinese Medicine, Jinan, Shandong Province, 250014, China; Department of Gastroenterology, Affiliated Hospital of Shandong University of Traditional Chinese Medicine, Jinan, Shandong Province, 250014, China; Department of Cardiovascular, Affiliated Hospital of Shandong University of Traditional Chinese Medicine, Jinan, Shandong Province, 250014, China; Department of Critical Care Medicine, Affiliated Hospital of Shandong University of Traditional Chinese Medicine, Jinan, Shandong Province, 250014, China; Affiliated Hospital of Shandong University of Traditional Chinese Medicine, Jinan, China; First School of Clinical Medicine, Shandong University of Traditional Chinese Medicine, Jinan, Shandong Province, 250014, China

**Keywords:** novel coronavirus, novel coronavirus infection, long COVID, sequelae, cardiovascular system

## Abstract

**Background:**

COVID-19 sequelae are long-term symptoms of COVID-19. Cardiovascular disease is not only a risk factor for the occurrence of COVID-19 sequelae but also a potential result directly or indirectly caused by COVID-19 infection.

**Objectives:**

The aim of this study is to investigate the cardiovascular system-related symptoms of outpatients and inpatients of the Cardiovascular Department of the Affiliated Hospital of Shandong University of Traditional Chinese Medicine after recovery from novel coronavirus infection, analyze the influencing factors, and symptom characteristics of related symptoms, and thereby provide a basis for further formulating a reasonable diagnosis and treatment plan.

**Materials and methods:**

From January 15, 2023 to February 15, 2023, 452 recovered patients with novel coronavirus infection who were admitted to the Cardiovascular Department of the Affiliated Hospital of Shandong University of Traditional Chinese Medicine due to symptoms of the cardiovascular system (complaints of chest pain and palpitations) were involved in this study. A unified questionnaire was used to record the general information, past medical history, characteristics of chest pain or palpitations, and other COVID-19-related sequelae of the selected patients. All data were statistically analyzed by SPSS 26.0 statistical software.

**Results:**

A total of 226 patients with cardiovascular symptoms and 226 patients without cardiovascular symptoms were included in this study. After univariate and multivariate logistic regression analysis, women (OR 2.081, 95% CI = 1.358–3.189) and young people (OR 2.557, 95% CI = 1.44–4.54) had a higher risk of cardiovascular symptoms; prehypertension (OR 1.905, 95% CI = 1.091–3.329) and hypertension (OR 2.287, 95% CI = 1.433–3.649) increased the risk of cardiovascular symptoms; patients with history of previous cardiovascular disease (OR 1.862, 95% CI = 1.16–2.988) and history of diabetes (OR 2.138, 95% CI = 1.058–4.319) had a higher risk of developing cardiovascular symptoms. The main symptoms related to COVID-19 sequelae reported by all 452 patients were fatigue (76.8%), shortness of breath (54.2%), dry mouth and bitter mouth (46.0%), gastrointestinal symptoms (42.7%), sleep disturbances (37.4%), sweating (31.9%), chills (29%), dizziness (25.7%), confusion of brain fog (25.2%), and tinnitus (14.6%). Compared with patients without cardiovascular symptoms, patients with cardiovascular symptoms were more likely to have shortness of breath (OR 3.521, 95% CI = 2.226–5.472), gastrointestinal symptoms (OR 2.039, 95% CI = 1.226–3.393), and dry mouth and bitter mouth (OR 1.918, 95% CI = 1.229–2.992). The differences were statistically significant (*P* < 0.05).

**Conclusion:**

In this new coronavirus infection, women, young people, the elderly, people with prehypertension, hypertension, and patients with a history of cardiovascular disease and diabetes have a higher risk of developing cardiovascular symptoms, and patients with cardiovascular symptoms are more likely to develop other COVID-19 sequelae.

## 1 Introduction

COVID-19 sequelae, also known as “long COVID-19,” are long-term symptoms of COVID-19. According to the clinical case definition published by the World Health Organization in October 2021, COVID-19 sequelae usually occur within 3 months after the onset of COVID-19 infection. They cannot be explained by other diagnoses. From the end of 2022 to the beginning of 2023, China experienced the peak period of COVID-19 infection. More and more studies and evidence show that most patients with COVID-19 recovery have sequelae of different systems and degrees, which may be new symptoms after recovery from infection, or the persistence of specific symptoms during acute infection. Cardiovascular disease is not only a risk factor for the occurrence of COVID-19 sequelae but also a potential result directly or indirectly caused by COVID-19 infection. It was found clinically that most patients who visited the Cardiovascular Department due to the sequelae of COVID-19 complained of chest pain and palpitations. This study collected the general information and prominent symptoms of relevant patients and systematically summarizes the relevant information of the patients to clarify the main manifestations of the sequelae of COVID-19 in the cardiovascular system, explore the influencing factors of related symptoms, and aim to raise people’s attention to the sequelae of COVID-19, to provide a basis for further formulating a reasonable treatment plan.

## 2 Materials and methods

### 2.1 Study design and setting

From January 15, 2023 to February 15, 2023, 452 patients in the outpatient department and ward of the Cardiovascular Department of the Affiliated Hospital of Shandong University of Traditional Chinese Medicine were involved in this study.

### 2.2 Participants

The inclusion criteria of participants are over 18 years and should have a negative COVID-19 test result after being infected with the new coronavirus.

### 2.3 Experimental procedure

A unified questionnaire was used for data collection. The questionnaire contents include general information such as age, sex, height, weight, blood pressure, smoking history, drinking history, and past medical history (cardiovascular disease, cerebrovascular disease, thyroid disease, diabetes, and dyslipidemia). The description of symptoms for both chest pain and palpitations included frequency, duration, and their nature.

### 2.4 Statistical analyses

For the statistical evaluation of the data, SPSS version 26.0 was utilized. Continuous variables that followed a normal distribution were presented as mean value ± standard deviation (SD), and comparisons between groups were made using the independent samples *t*-test. Non-normally distributed continuous variables were described using medians and interquartile ranges (IQR; P50 [P25, P75]), with the Wilcoxon rank-sum test applied for intergroup comparisons. Categorical variables were expressed as counts and percentages, and were analyzed using the Chi-square test or Fisher’s exact test as appropriate.

To identify factors associated with cardiovascular symptoms post-COVID-19, univariate logistic regression analyses were initially conducted. Variables with a *P*-value of less than 0.1 in the univariate analysis were then included in a multivariate logistic regression model. A stepwise selection method was employed to determine the final model, with entry and removal significance levels set at 0.05 and 0.10, respectively. The results of the multivariate analysis were reported as adjusted odds ratios (ORs) with corresponding 95% confidence intervals (CIs).

The statistical significance threshold was set at a *P*-value of less than 0.05. It is important to note that while statistical significance can suggest associations, it does not confirm causation, and the results should be interpreted within the context of the study’s limitations, including its single-center design and the potential for residual confounding.


**Ethical approval:** This study obtained approval from the Hospital Ethics Committee, and informed consent were obtained from all patients who participated in the study.

## 3 Results

### 3.1 Baseline data of the group with and without cardiovascular symptoms

A total of 452 patients aged 18–79 years, including 226 cases with cardiovascular symptoms and 226 cases without cardiovascular symptoms were involved in this study. Compared with the asymptomatic group, the proportion of women in the symptomatic group was higher, the blood pressure level was higher, the proportion of drinking history was lower, and the proportion of previous cardiovascular disease and diabetes was higher. The differences were statistically significant (*P* < 0.05) (Table 1).

**Table 1:** Demographics and clinical characteristics of the included patients

	Patients with cardiovascular symptoms	Patients without cardiovascular symptoms	*P* value	*X* ^2^/*Z* value (*t* value)
No. (%)	226 (50)	226 (50)		
Age, mean (SD), years	52.2	54.1	0.101	−1.643
Median (IQR), years	54 (47,59)	56 (45,63)		
Age by category, %	45 (19.9)	55 (24.3)	0.169	−1.374
Young adult				
Middle age	127 (56.2)	88 (38.9)		
Old age	54 (23.9)	83 (36.7)		
Female, %	131 (58.0)	154 (68.1)	0.025	−2.239
Male, %	95 (42.0)	72 (31.9)		
BMI, mean (SD)	24.8	24.5	0.434	0.784
BMI by category, %	2 (0.9)	8 (3.5)	0.760	−0.306
Underweight (<18.5)				
Normal (18.5 to <25)	126 (55.8)	118 (52.2)		
Overweight (25 to <30)	85 (37.6)	89 (39.4)		
Obese (30 to <40)	13 (5.8)	11 (4.9)		
Systolic pressure, mean (mmHg)	120.9	124.3	0.015	−2.453
Diastolic pressure, mean (mmHg)	78.1	77.6	0.455	0.650
Normal blood pressure	122 (54.0)	83 (36.7)	0.000	−3.779
Prehypertension	35 (15.5)	40 (17.7)		
Hypertension	69 (30.5)	103 (45.6)		
Heart rate, mean (times/min)	73	74	0.413	−0.818
Former and current smokers	36 (15.9)	37 (16.4)	0.898	−0.128
Former and current drinkers	69 (30.5)	42 (18.6)	0.003	−2.947
Any pre-existing chronic condition	50 (22.1)	88 (38.9)	0.000	−3.877
Heart disease				
Diabetes	14 (6.2)	33 (14.6)	0.003	−2.925
Dyslipidemia	31 (13.7)	41 (18.1)	0.199	−1.639
Cerebrovascular disease	10 (4.4)	17 (7.5)	0.162	−1.400
Thyroid disease	31 (13.7)	46 (20.4)	0.061	−1.875
Doses of vaccination	48 (21.2)	42 (18.6)	0.480	−0.706
Less than 3 doses				
Greater or equal than 3 doses	178 (78.8)	184 (81.4)		

### 3.2 Other symptoms associated with COVID-19 sequelae

The main symptoms related to COVID-19 sequelae reported by 452 patients were fatigue (76.8%), shortness of breath (54.2%), dry mouth and bitter mouth (46.0%), gastrointestinal symptoms (42.7%), sleep disturbances (37.4%), sweating (31.9%), chills (29%), dizziness (25.7%), confusion or brain fog (25.2%), and tinnitus (14.6%) (Table 2).

**Table 2:** Symptoms of COVID-19 sequalae

	All patients (*n* = 452)	Patients with cardiovascular symptoms (*n* = 226)	Patients without cardiovascular symptoms (*n* = 226)	*P* value	OR (95% CI)
**Symptom, N (%)**					
Shortness of breath	245 (54.2)	83 (36.7)	162 (71.7)	0.000*	3.521 (2.226, 5.472)
Fatigue	347 (76.8)	155 (68.6)	192 (85.0)	0.179	1.438 (0.846, 2.446)
Dizziness	116 (25.7)	37 (16.4)	79 (35.0)	0.006*	2.039 (1.226, 3.393)
Chills	131 (29.0)	51 (22.6)	71 (31.4)	0.650	1.116 (0.696, 1.789)
Sweating	144 (31.9)	70 (31.0)	74 (32.7)	0.079	0.659 (0.414, 1.049)
Gastrointestinal symptoms	193 (42.7)	71 (31.4)	94 (41.6)	0.002*	2.033 (1.301, 3.179)
Dry mouth and bitter mouth	208 (46.0)	77 (34.1)	131 (58.0)	0.004*	1.918 (1.229, 2.992)
Confusion or brain fog	114 (25.2)	49 (21.7)	65 (28.8)	0.671	0.896 (0.541, 1.458)
Sleep disturbances	169 (37.4)	71 (31.4)	98 (43.4)	0.517	0.856 (0.535, 1.369)
Tinnitus	66 (14.6)	22 (9.7)	44 (19.5)	0.465	1.264 (0.657, 2.369)

*means *p* < 0.01.

Compared with patients without cardiovascular symptoms, patients with cardiovascular symptoms were more likely to be accompanied by shortness of breath (OR 3.521, 95% CI = 2.226–5.472), dizziness (OR 2.039, 95% CI = 1.226–3.393), gastrointestinal symptoms (OR 2.033, 95% CI = 1.301–3.179), and dry mouth and bitter mouth (OR 1.918, 95% CI = 1.229–2.992). The differences were statistically significant (*P* < 0.01) (Table 2).

### 3.3 Analysis of influencing factors of cardiovascular symptoms

In the assessment of cardiovascular symptoms post-COVID-19, a comprehensive statistical approach was employed to discern the relationship between various patient characteristics and the manifestation of cardiovascular sequelae. The dependent variable in this analysis was the presence of cardiovascular symptoms, coded as binary outcomes where “yes” was assigned a value of 1 and “no” a value of 0. A range of independent variables was considered, including age classification, gender, body mass index (BMI) classification, blood pressure level classification, resting heart rate, and medical history encompassing cardiovascular disease, cerebrovascular disease, diabetes, and thyroid disease. Lifestyle factors such as smoking and drinking history were also incorporated into the analysis.

To evaluate the potential influence of these variables on the occurrence of cardiovascular sequelae, both univariate and multivariate logistic regression models were applied. The univariate analysis served as a preliminary step, identifying variables with a *P*-value less than 0.1, which were then included in the subsequent multivariate model. This selection criterion ensured that only variables with a potential association were considered for more complex analysis, thereby refining the model and enhancing the reliability of the results.

The multivariate logistic regression analysis utilized the forward LR method, a forward stepwise regression approach based on maximum likelihood estimation. This technique iteratively selects the most statistically significant variables, adding them to the model one at a time, and removing any that do not contribute to the model’s predictive power. This process is governed by predefined significance levels for entry and removal from the model, set at 0.05 and 0.10, respectively.

**Figure 1:**
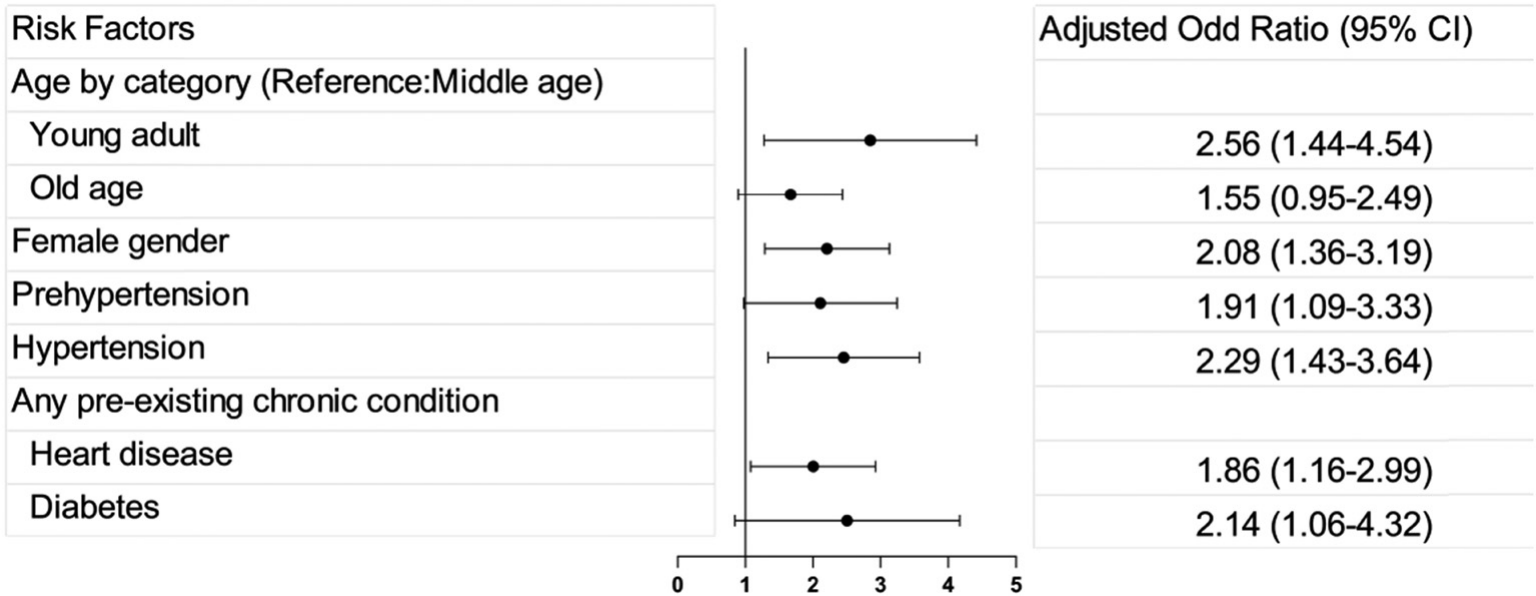
The forest plot for the regression analysis.

The findings from the multivariate logistic regression revealed that females exhibited a higher risk of developing cardiovascular symptoms post-COVID-19, with an OR of 2.081 and a 95% CI of 1.358–3.189. Similarly, young individuals were identified as a higher risk group, with an OR of 2.557 and a 95% CI of 1.44–4.54. Blood pressure levels were also significant predictors, with prehypertension and hypertension associated with increased risks of cardiovascular symptoms, as indicated by ORs of 1.905 (95% CI = 1.091–3.329) and 2.287 (95% CI = 1.433–3.649), respectively. Additionally, a history of cardiovascular disease and diabetes were found to be significant risk factors, with ORs of 1.862 (95% CI = 1.16–2.988) and 2.138 (95% CI = 1.058–4.319), respectively (Table 3 and Figure 1). These results were statistically significant, with *P*-values less than 0.05, suggesting a robust association between these factors and the development of cardiovascular symptoms post-COVID-19.

**Table 3:** Impact of gender, BMI, blood pressure, heart rate, medical history, smoking and drinking history, and full vaccination before COVID-19 infection on the occurrence of cardiovascular sequelae

	Patients without cardiovascular symptoms (*n* = 226)	Patients with cardiovascular symptoms (*n* = 226)	*P* value	Adjust-ed OR	95% CI	95% CI
**Age by category**						
Young adult	45 (19.9)	55 (24.3)	0.001	2.557	1.44	4.54
Middle age	127 (56.2)	88 (38.9)	Ref.			
Old age	54 (23.9)	83 (36.7)	0.074	1.545	0.959	2.49
Sex (female)	131 (58.0)	154 (68.1)	0.001	2.081	1.358	3.189
**Blood pressure by classification**						
Normal blood pressure	122 (54.0)	83 (36.7)	Ref.			
Prehypertension	35 (15.5)	40 (17.7)	0.024	1.905	1.091	3.329
Hypertension	69 (30.5)	103 (45.6)	0.001	2.287	1.433	3.649
**Any pre-existing chronic condition**						
Heart disease	50 (22.1)	88 (38.9)	0.01	1.862	1.16	2.988
Diabetes	14 (6.2)	33 (14.6)	0.034	2.138	1.058	4.319

### 3.4 Analysis of factors affecting the severity of cardiovascular symptoms

#### 3.4.1 Chest pain severity

The frequency of chest pain (“≤1 time per day” = 1, “1 to 3 times a day” = 2, “>4 times a day” = 3) and the duration of chest pain (“<5 min each time” = 1, “every time 5 to 10 min” = 2, “every time > 10 min” = 3) as the dependent variables, with age classification, gender, BMI classification, blood pressure level classification, resting heart rate, smoking history, drinking history, history of cardiovascular disease, history of cerebrovascular disease, history of diabetes, and history of thyroid disease were used as independent variables and included in an ordered multiclass logistic regression model. The results showed that patients with a history of drinking reduced the risk of increased chest pain frequency by 74.3% and reduced the risk of increased chest pain duration by 68.5% (*P* < 0.05) (Tables 4 and 5).

**Table 4:** Influencing factors of chest pain frequency

Everyday chest pain frequency	*P* value	OR (95% CI)
With drinking history	0.008	0.257 (0.095, 0.687)
Without drinking history	Ref.	

**Table 5:** Influencing factors of chest pain duration

Chest pain duration	*P* value	OR (95% CI)
With drinking history	0.041	0.325 (0.111, 0.954)
Without drinking history	Ref.	

#### 3.4.2 Palpitations severity

The frequency of palpitations (“≤1 time per day” = 1, “1 to 3 times a day” = 2, “>4 times a day” = 3) and the duration of palpitations (“every time < 1 h = 1, “every 1 to 12 h” = 2, “each time 12 to 24 h” = 3, each time > 24 h = 4) as the dependent variable, with age classification, gender, BMI classification, blood pressure level classification, resting heart rate, smoking history, drinking history, history of cardiovascular disease, history of cerebrovascular disease, history of diabetes, and history of thyroid disease were used as independent variables and included in the ordered multiclass logistic regression model. The results showed that in patients without previous cardiovascular disease, the risk of increased palpitation times decreased by 75.8%. In obese patients, the risk of palpitations lasting longer increased 2.24 times. 92.8% of patients had paroxysmal palpitations, and 7.2% of patients had persistent palpitations or arrhythmias (Tables 6 and 7).

**Table 6:** Influencing factors of the palpitations frequency

Everyday palpitations frequency	*P* value	OR (95% CI)
With cardiovascular disease history	Ref.	
Without cardiovascular disease history	0.008	0.342 (0.155, 0.755)

**Table 7:** Influencing factors of palpitations duration

Everyday palpitations duration	*P* value	OR (95% CI)
Underweight	0.712	
Normal weight	Ref.	
Overweight	0.682	
Obese	0.007	2.240 (2.138, 127.868)

## 4 Discussion

The present study conducted at the Affiliated Hospital of Shandong University of Traditional Chinese Medicine offers novel insights into the post-COVID-19 cardiovascular sequelae, revealing significant associations with demographic and pre-existing health conditions. Our findings underscore the heightened vulnerability of certain populations – particularly women, younger individuals, and those with pre-existing cardiovascular conditions – to the development of cardiovascular symptoms following COVID-19 recovery. This study is among the first to delineate the risk profile for cardiovascular sequelae in a post-COVID-19 cohort within a traditional Chinese medicine hospital setting, providing a unique perspective on the intersection of viral infection aftermath and cardiovascular health.

Heart discomfort is a common symptom of patients with COVID-19, mainly including chest pain, palpitations, fatigue, shortness of breath, etc. Previous studies have shown that the new coronavirus will cause damage to the cardiovascular system, may impact the heart, and even cause serious complications such as myocarditis and myocardial infarction. The mechanism is mainly related to myocardial injury. The first cause is that the virus directly invades the cells by binding to the angiotensin-converting enzyme 2 receptor on the surface of myocardial cells, the second is a myocardial injury caused by an immune inflammatory response and cytokine storm, and the third is an ischemic myocardial injury caused by cellular hypoxia. A cohort study involving 12,095,836 cases found that even people without high-risk factors for cardiovascular disease still had an increased risk of cardiovascular disease after infection with the new coronavirus. Paying attention to the impact of the new coronavirus on the heart and studying the risk factors of related sequelae are crucial for predicting the occurrence of sequelae and improving the quality of life of patients.

Our analysis indicates that young adults, a demographic typically perceived as having a lower risk for severe COVID-19 outcomes, are paradoxically at an elevated risk for cardiovascular sequelae. This unexpected finding challenges the prevailing understanding of COVID-19’s impact across age groups and suggests that factors such as lifestyle, stress, and possibly vaccination status may contribute to this increased susceptibility. Existing studies have shown that aging is an independent risk factor for COVID-19. Electronic health record analysis of age and long-term diagnosis of new coronavirus shows that the long-term risk of COVID-19 virus is associated with age in an inverted U shape. The risk of 45–69 years old and 55–69 years old are the highest; the risk was not higher among those aged 80 years and older than the reference population aged 18–24 [1]. This study found that young people have a higher risk of cardiovascular sequelae than middle-aged and elderly after being infected with the COVID-19. In addition, high work pressure, eating disorders, and bad living habits of young people may also be influencing factors. Studies have observed that vaccination with the second dose of mRNA-1273 vaccine may increase the probability of myocarditis in young people, so the higher risk of cardiovascular sequelae in young people may be related to vaccination [2]. In addition, Osmanov et al. believe that in children and adolescents, immune mechanisms may be responsible for the increased risk of long-term consequences of infection, and the sequelae of COVID-19 may be associated with the immune responses of mast cell activation syndrome and helper T cell type 2 in children with allergic diseases [3].

Moreover, the study’s revelation that women are disproportionately affected by long-term cardiovascular symptoms post-COVID-19 adds to the growing body of evidence on gender disparities in the pandemic’s aftermath. The potential role of immunological differences and hormonal influences in this gender-specific risk profile warrants further exploration. Studies have shown that there seem to be significant gender differences in long-term cardiovascular outcomes after COVID-19. Middle-aged women are twice as likely to suffer from long-term COVID-19 as men. The difference in inflammatory load between male and female populations may explain the gender differences in clinical manifestations and cardiovascular outcomes after COVID-19 [4]. Previous studies have also shown that women are associated with COVID-19 symptoms, especially persistent fatigue, anxiety, and depression. The reason may be that women have a stronger immune response. For example, women have a higher proportion of autoimmune diseases. It has been reported that women produce stronger immunoglobulin G antibodies in the early stage of the disease, which may lead to more favorable outcomes in women. Women recover more quickly from COVID-19 but may also play a role in perpetuating disease manifestations [5]. Even after recovery, estrogen in women may maintain the role played in the hyper-inflammatory state of the acute phase, which may also increase the risk of developing COVID-19 [6,7]. In addition, women are generally more concerned about their bodies and related pain, which may also be the reason why the survey shows that women are at higher risk of developing COVID-19 [8].

The study also reaffirms the critical influence of pre-existing cardiovascular conditions on the severity and incidence of post-COVID-19 cardiovascular symptoms. Patients with previous cardiovascular diseases are more likely to be infected with the new coronavirus. These findings emphasize the need for targeted monitoring and management strategies for patients with cardiometabolic disorders in the context of COVID-19 recovery. The virus has long-term potential effects on heart damage and is more likely to have related symptoms such as palpitations or chest pain. Patients with existing cardiometabolic diseases may have a higher risk of developing an acute state after infection with the new coronavirus, accompanied by complications and significantly affecting the prognosis [9]. On the other hand, COVID-19 itself may aggravate cardiac damage. It has been reported that previous history of coronary heart disease and elevated cardiac troponin I levels are two independent determinants of clinical status in COVID-19 patients [10]. Elderly patients with coronary heart disease, hypertension, diabetes, and chronic kidney disease are more likely to develop severe sequelae after COVID-19. Transthoracic echocardiogram imaging showed regional wall motion abnormalities, left or right ventricle abnormalities, systolic dysfunction, diastolic dysfunction, and pericardial effusion in the acute phase of COVID-19 [11]. In patients diagnosed with various heart diseases, COVID-19 infection may be one of the triggers for exacerbation and death [12]; patients with new-onset myocardial dysfunction, inflammation, or cardiovascular magnetic resonance scarring after COVID-19 may have increased risk of cardiac failure or recurrence of arrhythmia [13]. Patients with hyperglycemia have metabolic abnormalities, and patients with metabolic abnormalities have a higher risk of severe COVID-19 and COVID-19 sequelae. Obesity is a risk factor for cardiovascular disease, and the possible mechanisms include sympathetic nerve activation, endothelial dysfunction, oxidative stress, rheumatoid arthritis activation, metabolic dysfunction, etc., which can affect hemodynamics and cardiac structure, thereby increasing palpitations and the risk of developing related symptoms.

Interestingly, our data suggest a protective effect of alcohol consumption against the severity of chest pain, a finding that contrasts with the established role of alcohol as a risk factor for cardiovascular disease. This counterintuitive association may reflect complex interactions between alcohol intake, cardiovascular health, and COVID-19, which merit further investigation. It is imperative to approach these results with caution, considering the potential confounding factors and the need for larger-scale studies to validate the observed relationship. Alcohol consumption is a well-recognized risk factor for cardiovascular disease. Statistical analysis of the data in this study found that a history of alcohol consumption reduced the risk of increased chest pain severity. However, the small sample size may lead to statistical bias in the data, hence, no conclusion can be drawn. Additionally, the study demonstrated a J-shaped relationship between alcohol consumption and cardiovascular disease incidence, total mortality, and cardiovascular disease mortality in multivariate models. In a multivariable model, alcohol intake of 5–14.9 g per day was associated with a 26, 35, and 51% lower risk of cardiovascular disease, total mortality, and cardiovascular disease mortality, respectively, compared with abstainers. The mechanism may be related to the effect of alcohol on lipids and insulin sensitivity [14]; there is also evidence that alcohol consumption is positively associated with coronary artery disease, atrial fibrillation, and abdominal aortic aneurysm, but this association is attenuated after adjustment for smoking, hence smoking may have some effect in that study [15]; alcohol abuse is associated with increased odds of clinically high serum total cholesterol and triglyceride levels [16], but a short-term intervention study showed that moderate alcohol consumption reduced several cardiovascular biomarkers including high-density lipoprotein, adiponectin levels, and fibrinogen [17]. Although some studies in the past have shown that light drinking may have a protective effect on ischemic heart disease in women, the irreversible damage caused by alcohol to the cardiovascular system has offset this protective effect. The relationship between drinking history and drinking volume with COVID-19 still needs further research.

The implications of these findings are profound, highlighting the necessity for a nuanced approach to post-COVID-19 care that accounts for the diverse risk factors influencing cardiovascular sequelae. Healthcare providers should be cognizant of these risk factors when evaluating and managing patients in the post-recovery phase. Additionally, our study calls for increased vigilance in the follow-up of young adults and women who have recovered from COVID-19, as they may harbor a higher risk for long-term cardiovascular complications.

### 4.1 Limitations of the study

There are a few limitations in this study. First, this study only involved patients from a single center. The sample size was small, so there was a statistical bias. Second, most symptoms are subjective and easily affected by subjective bias. Third, this study lacks the assessment of the patients’ symptoms before and during the infection of COVID-19. A comparative analysis of the patients’ symptoms before and after the COVID-19 infection will be of great significance in evaluating the impact of the COVID-19 sequelae on various systems. Finally, although this study has made statistics on the sequelae of COVID-19, it lacked quantitative and specific indicators. For example, “fatigue” is one of the main sequelae. If relevant scales are applied for objective and comparative analysis, the results can be more persuasive.

## 5 Conclusion

The study conducted at the Affiliated Hospital of Shandong University of Traditional Chinese Medicine provides insights into the prevalence and risk factors associated with cardiovascular symptoms in patients who have recovered from COVID-19. Our findings suggest a correlation between certain demographic and health-related factors and the increased likelihood of experiencing cardiovascular symptoms post-COVID-19 infection.

It was observed that women, younger individuals, and the elderly exhibited a higher propensity for cardiovascular symptoms. Moreover, pre-existing conditions such as prehypertension, hypertension, cardiovascular diseases, and diabetes were identified as contributing factors to the increased risk of cardiovascular sequelae. These associations underscore the importance of vigilant monitoring and management of cardiovascular health, particularly in these identified higher-risk groups, during the post-recovery phase of COVID-19.

Additionally, the study revealed that individuals with cardiovascular symptoms post-COVID-19 were more prone to experiencing a range of other sequelae, including fatigue, shortness of breath, and gastrointestinal disturbances. This finding indicates a potential interconnection between cardiovascular symptoms and other post-COVID-19 conditions, which warrants further investigation to understand the underlying mechanisms and to develop comprehensive management strategies.

While the study provides valuable information, it is important to acknowledge its limitations, including the single-center design and the reliance on self-reported symptoms, which may introduce subjective bias. Future research with larger, multi-center cohorts and objective diagnostic measures is recommended to validate these findings and to explore the long-term implications of COVID-19 on cardiovascular health.

In conclusion, our study contributes to the growing body of evidence on post-COVID-19 sequelae and highlights the need for targeted follow-up care for patients at higher risk of cardiovascular complications. Healthcare providers should be aware of these risk factors when assessing and treating patients who have recovered from COVID-19, to mitigate the potential long-term health impacts of the disease.
